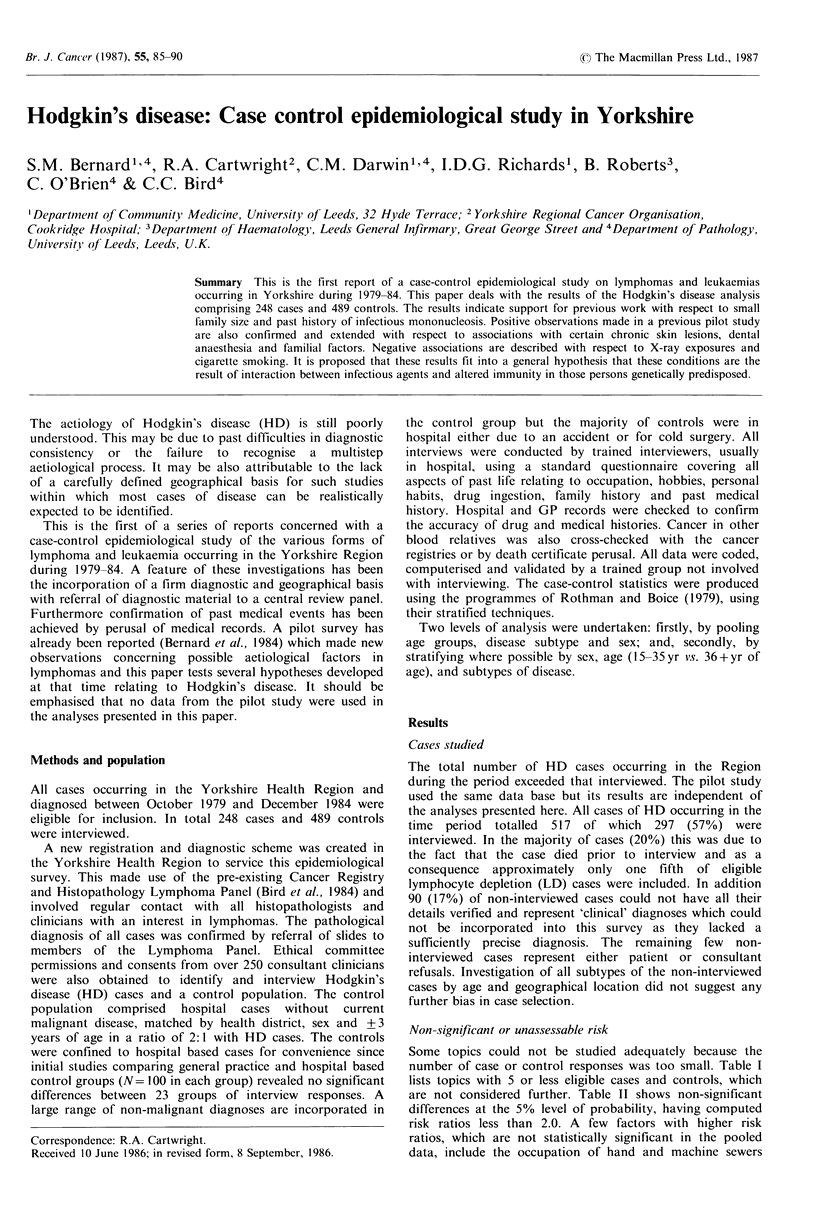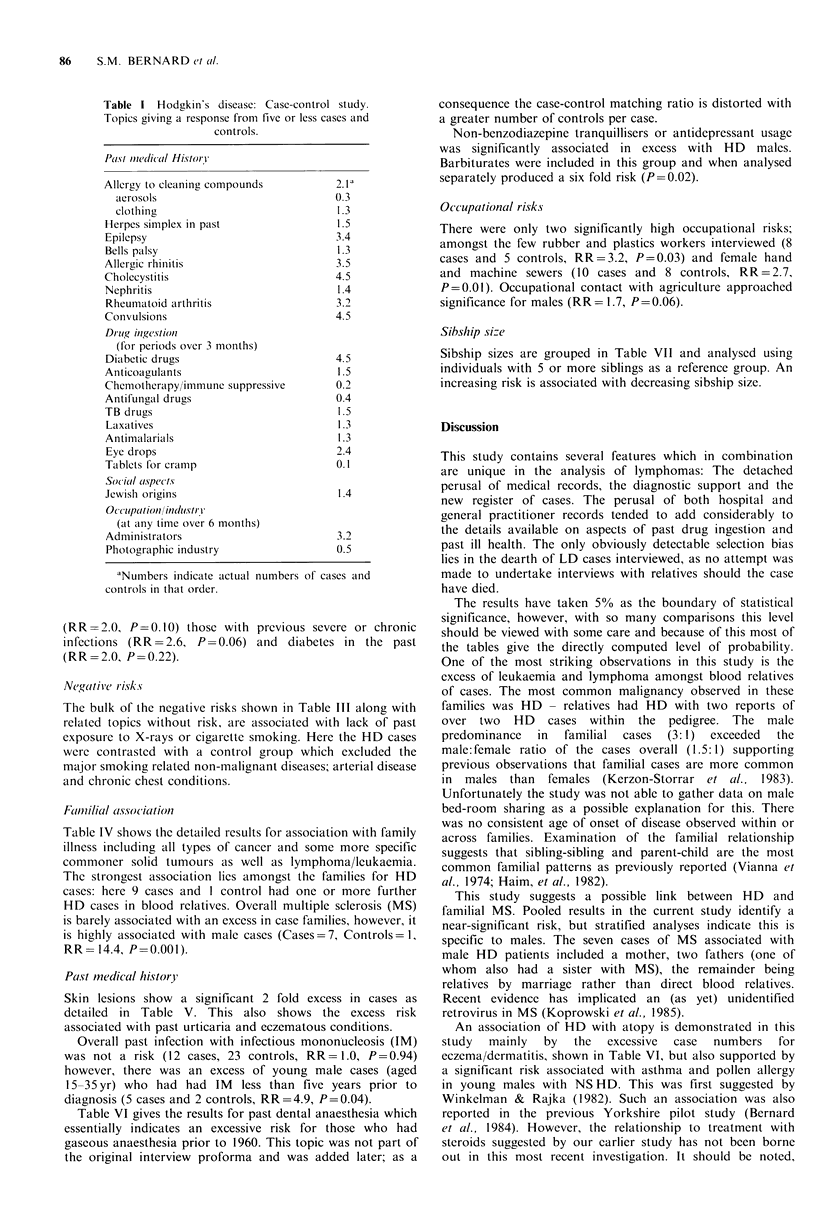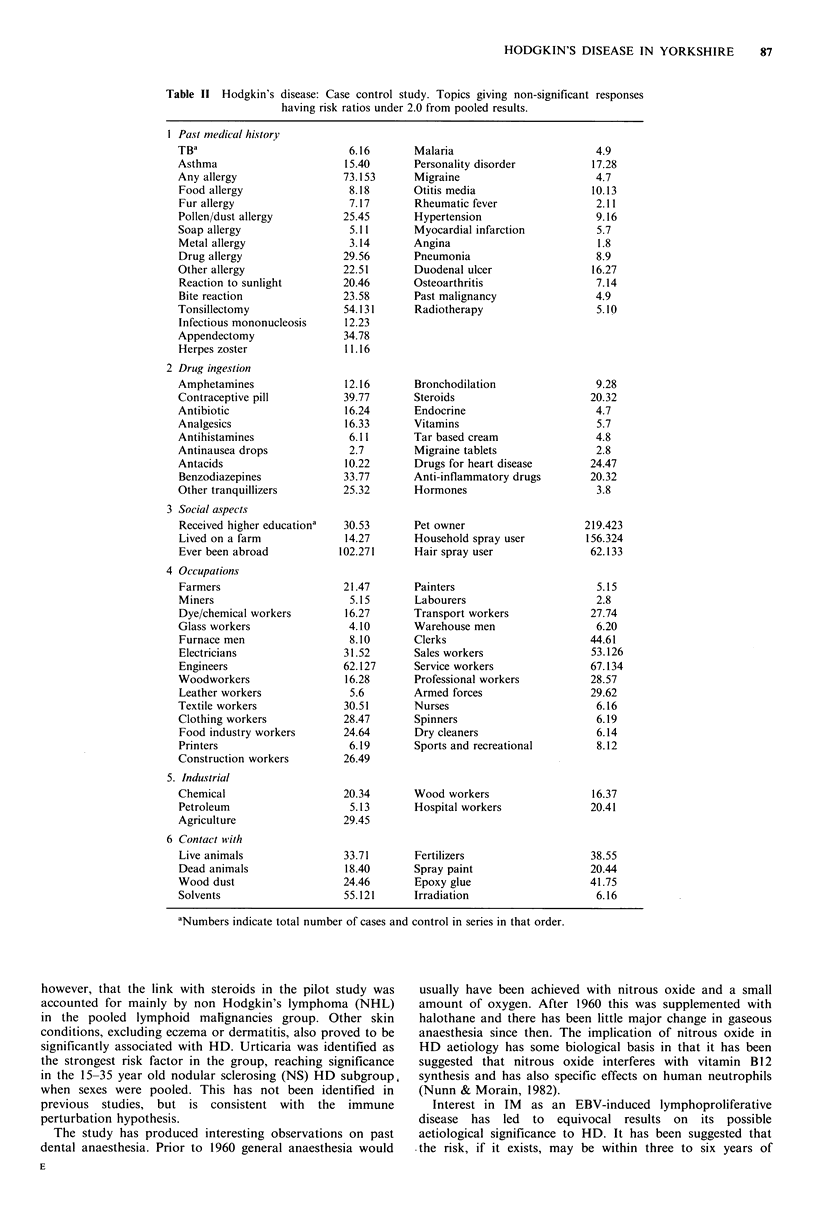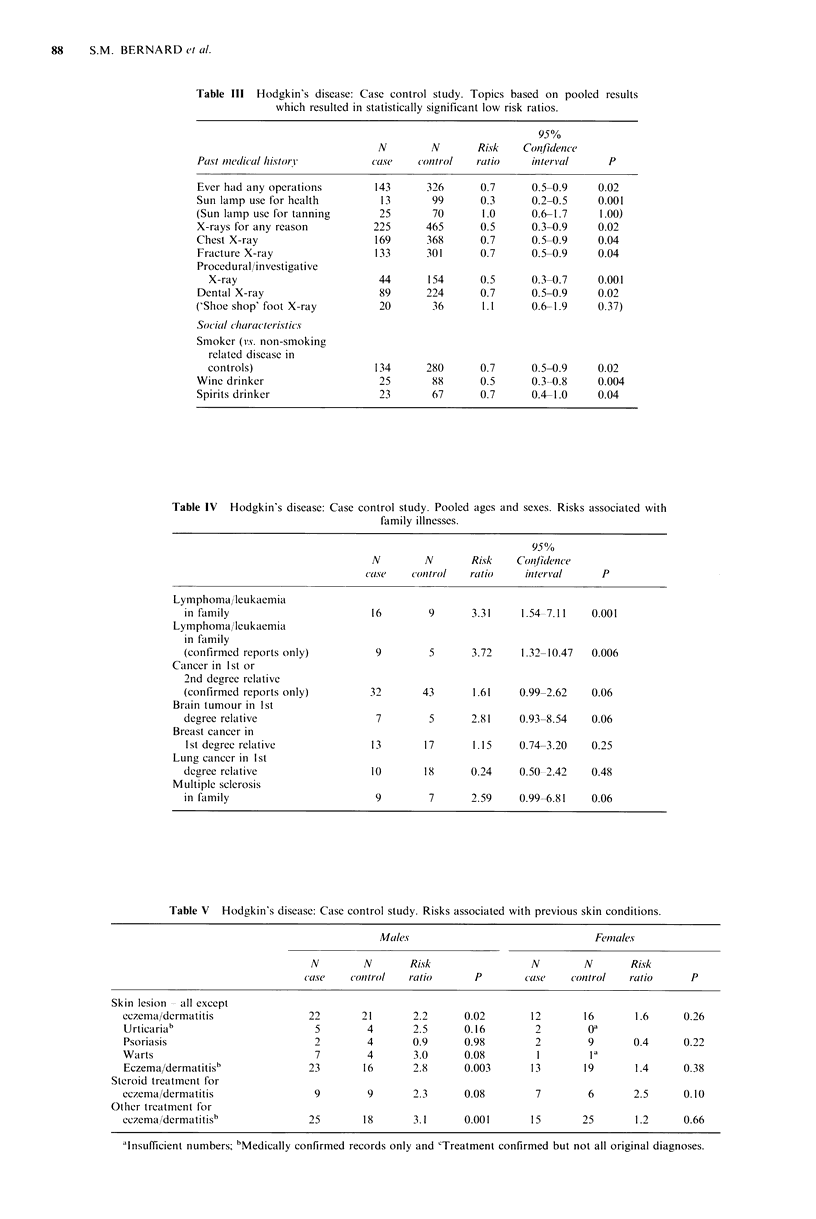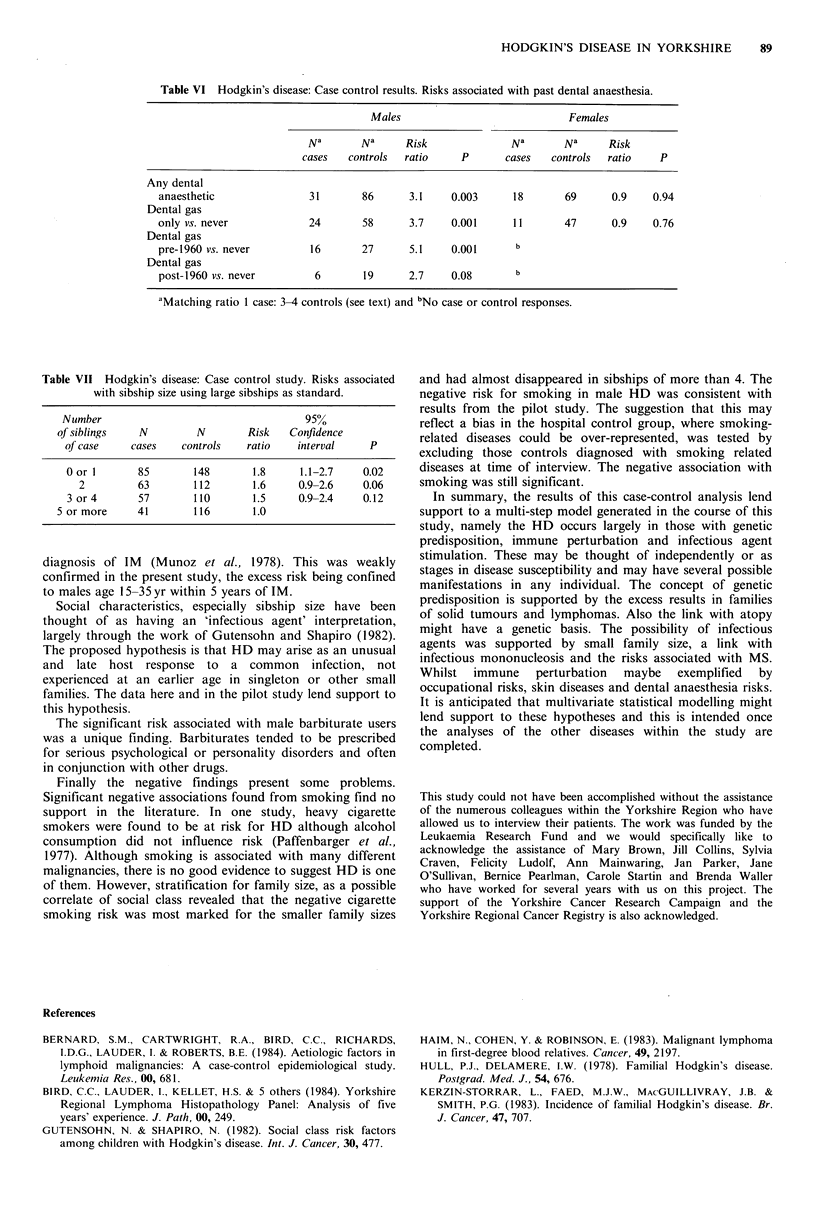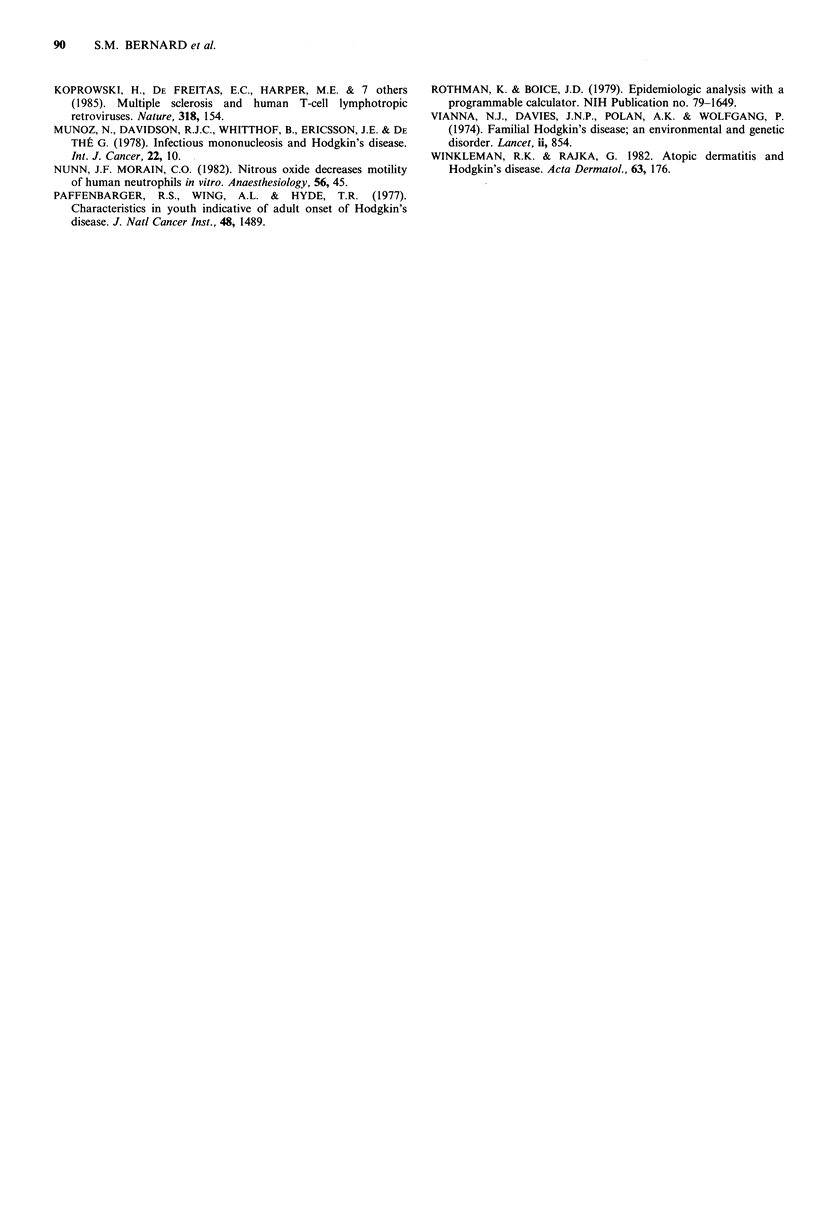# Hodgkin's disease: case control epidemiological study in Yorkshire.

**DOI:** 10.1038/bjc.1987.18

**Published:** 1987-01

**Authors:** S. M. Bernard, R. A. Cartwright, C. M. Darwin, I. D. Richards, B. Roberts, C. O'Brien, C. C. Bird

## Abstract

This is the first report of a case-control epidemiological study on lymphomas and leukaemias occurring in Yorkshire during 1979-84. This paper deals with the results of the Hodgkin's disease analysis comprising 248 cases and 489 controls. The results indicate support for previous work with respect to small family size and past history of infectious mononucleosis. Positive observations made in a previous pilot study are also confirmed and extended with respect to associations with certain chronic skin lesions, dental anaesthesia and familial factors. Negative associations are described with respect to X-ray exposures and cigarette smoking. It is proposed that these results fit into a general hypothesis that these conditions are the result of interaction between infectious agents and altered immunity in those persons genetically predisposed.


Br. J. Cancer (1987), 55, 85-90 © The Macmillan Press Ltd., 1987

### Hodgkin's disease: Case control epidemiological study in Yorkshire

S.M. Bernard[1,4], R.A. Cartwright[2], C.M. Darwin[1,4], I.D.G. Richards[1], B. Roberts[3], C. O'Brien[4] & C.C. Bird[4]

[1]*Department of Community Medicine, University of Leeds, 32 Hyde Terrace;* [2]*Yorkshire Regional Cancer Organisation, Cookridge Hospital;* [3]*Department of Haematology, Leeds General Infirmary, Great George Street and* [4]*Department of Pathology, University of Leeds, Leeds, U.K.*

**Summary** This is the first report of a case-control epidemiological study on lymphomas and leukaemias occurring in Yorkshire during 1979–84. This paper deals with the results of the Hodgkin's disease analysis comprising 248 cases and 489 controls. The results indicate support for previous work with respect to small family size and past history of infectious mononucleosis. Positive observations made in a previous pilot study are also confirmed and extended with respect to associations with certain chronic skin lesions, dental anaesthesia and familial factors. Negative associations are described with respect to X-ray exposures and cigarette smoking. It is proposed that these results fit into a general hypothesis that these conditions are the result of interaction between infectious agents and altered immunity in those persons genetically predisposed.

The aetiology of Hodgkin's disease (HD) is still poorly understood. This may be due to past difficulties in diagnostic consistency or the failure to recognise a multistep aetiological process. It may be also attributable to the lack of a carefully defined geographical basis for such studies within which most cases of disease can be realistically expected to be identified.

This is the first of a series of reports concerned with a case-control epidemiological study of the various forms of lymphoma and leukaemia occurring in the Yorkshire Region during 1979–84. A feature of these investigations has been the incorporation of a firm diagnostic and geographical basis with referral of diagnostic material to a central review panel. Furthermore confirmation of past medical events has been achieved by perusal of medical records. A pilot survey has already been reported (Bernard *et al.*, 1984) which made new observations concerning possible aetiological factors in lymphomas and this paper tests several hypotheses developed at that time relating to Hodgkin's disease. It should be emphasised that no data from the pilot study were used in the analyses presented in this paper.

### Methods and population

All cases occurring in the Yorkshire Health Region and diagnosed between October 1979 and December 1984 were eligible for inclusion. In total 248 cases and 489 controls were interviewed.

A new registration and diagnostic scheme was created in the Yorkshire Health Region to service this epidemiological survey. This made use of the pre-existing Cancer Registry and Histopathology Lymphoma Panel (Bird *et al.*, 1984) and involved regular contact with all histopathologists and clinicians with an interest in lymphomas. The pathological diagnosis of all cases was confirmed by referral of slides to members of the Lymphoma Panel. Ethical committee permissions and consents from over 250 consultant clinicians were also obtained to identify and interview Hodgkin's disease (HD) cases and a control population. The control population comprised hospital cases without current malignant disease, matched by health district, sex and $\pm 3$ years of age in a ratio of 2:1 with HD cases. The controls were confined to hospital based cases for convenience since initial studies comparing general practice and hospital based control groups ($N=100$ in each group) revealed no significant differences between 23 groups of interview responses. A large range of non-malignant diagnoses are incorporated in the control group but the majority of controls were in hospital either due to an accident or for cold surgery. All interviews were conducted by trained interviewers, usually in hospital, using a standard questionnaire covering all aspects of past life relating to occupation, hobbies, personal habits, drug ingestion, family history and past medical history. Hospital and GP records were checked to confirm the accuracy of drug and medical histories. Cancer in other blood relatives was also cross-checked with the cancer registries or by death certificate perusal. All data were coded, computerised and validated by a trained group not involved with interviewing. The case-control statistics were produced using the programmes of Rothman and Boice (1979), using their stratified techniques.

Two levels of analysis were undertaken: firstly, by pooling age groups, disease subtype and sex; and, secondly, by stratifying where possible by sex, age (15–35 yr *vs.* 36+yr of age), and subtypes of disease.

### Results

### Cases studied

The total number of HD cases occurring in the Region during the period exceeded that interviewed. The pilot study used the same data base but its results are independent of the analyses presented here. All cases of HD occurring in the time period totalled 517 of which 297 (57%) were interviewed. In the majority of cases (20%) this was due to the fact that the case died prior to interview and as a consequence approximately only one fifth of eligible lymphocyte depletion (LD) cases were included. In addition 90 (17%) of non-interviewed cases could not have all their details verified and represent 'clinical' diagnoses which could not be incorporated into this survey as they lacked a sufficiently precise diagnosis. The remaining few non-interviewed cases represent either patient or consultant refusals. Investigation of all subtypes of the non-interviewed cases by age and geographical location did not suggest any further bias in case selection.

### Non-significant or unassessable risk

Some topics could not be studied adequately because the number of case or control responses was too small. Table I lists topics with 5 or less eligible cases and controls, which are not considered further. Table II shows non-significant differences at the 5% level of probability, having computed risk ratios less than 2.0. A few factors with higher risk ratios, which are not statistically significant in the pooled data, include the occupation of hand and machine sewers

Correspondence: R.A. Cartwright.
Received 10 June 1986; in revised form, 8 September, 1986.

**Table I** Hodgkin's disease: Case-control study. Topics giving a response from five or less cases and controls.

| *Past medical History* | |
|---|---|
| Allergy to cleaning compounds | 2.1[a] |
| aerosols | 0.3 |
| clothing | 1.3 |
| Herpes simplex in past | 1.5 |
| Epilepsy | 3.4 |
| Bells palsy | 1.3 |
| Allergic rhinitis | 3.5 |
| Cholecystitis | 4.5 |
| Nephritis | 1.4 |
| Rheumatoid arthritis | 3.2 |
| Convulsions | 4.5 |
| *Drug ingestion* (for periods over 3 months) | |
| Diabetic drugs | 4.5 |
| Anticoagulants | 1.5 |
| Chemotherapy/immune suppressive | 0.2 |
| Antifungal drugs | 0.4 |
| TB drugs | 1.5 |
| Laxatives | 1.3 |
| Antimalarials | 1.3 |
| Eye drops | 2.4 |
| Tablets for cramp | 0.1 |
| *Social aspects* | |
| Jewish origins | 1.4 |
| *Occupation/industry* (at any time over 6 months) | |
| Administrators | 3.2 |
| Photographic industry | 0.5 |

[a]Numbers indicate actual numbers of cases and controls in that order.

(RR = 2.0, $P$ = 0.10) those with previous severe or chronic infections (RR = 2.6, $P$ = 0.06) and diabetes in the past (RR = 2.0, $P$ = 0.22).

*Negative risks*

The bulk of the negative risks shown in Table III along with related topics without risk, are associated with lack of past exposure to X-rays or cigarette smoking. Here the HD cases were contrasted with a control group which excluded the major smoking related non-malignant diseases; arterial disease and chronic chest conditions.

*Familial association*

Table IV shows the detailed results for association with family illness including all types of cancer and some more specific commoner solid tumours as well as lymphoma/leukaemia. The strongest association lies amongst the families for HD cases: here 9 cases and 1 control had one or more further HD cases in blood relatives. Overall multiple sclerosis (MS) is barely associated with an excess in case families, however, it is highly associated with male cases (Cases = 7, Controls = 1, RR = 14.4, $P$ = 0.001).

*Past medical history*

Skin lesions show a significant 2 fold excess in cases as detailed in Table V. This also shows the excess risk associated with past urticaria and eczematous conditions.

Overall past infection with infectious mononucleosis (IM) was not a risk (12 cases, 23 controls, RR = 1.0, $P$ = 0.94) however, there was an excess of young male cases (aged 15–35 yr) who had had IM less than five years prior to diagnosis (5 cases and 2 controls, RR = 4.9, $P$ = 0.04).

Table VI gives the results for past dental anaesthesia which essentially indicates an excessive risk for those who had gaseous anaesthesia prior to 1960. This topic was not part of the original interview proforma and was added later; as a consequence the case-control matching ratio is distorted with a greater number of controls per case.

Non-benzodiazepine tranquillisers or antidepressant usage was significantly associated in excess with HD males. Barbiturates were included in this group and when analysed separately produced a six fold risk ($P$ = 0.02).

*Occupational risks*

There were only two significantly high occupational risks; amongst the few rubber and plastics workers interviewed (8 cases and 5 controls, RR = 3.2, $P$ = 0.03) and female hand and machine sewers (10 cases and 8 controls, RR = 2.7, $P$ = 0.01). Occupational contact with agriculture approached significance for males (RR = 1.7, $P$ = 0.06).

*Sibship size*

Sibship sizes are grouped in Table VII and analysed using individuals with 5 or more siblings as a reference group. An increasing risk is associated with decreasing sibship size.

### Discussion

This study contains several features which in combination are unique in the analysis of lymphomas: The detached perusal of medical records, the diagnostic support and the new register of cases. The perusal of both hospital and general practitioner records tended to add considerably to the details available on aspects of past drug ingestion and past ill health. The only obviously detectable selection bias lies in the dearth of LD cases interviewed, as no attempt was made to undertake interviews with relatives should the case have died.

The results have taken 5% as the boundary of statistical significance, however, with so many comparisons this level should be viewed with some care and because of this most of the tables give the directly computed level of probability. One of the most striking observations in this study is the excess of leukaemia and lymphoma amongst blood relatives of cases. The most common malignancy observed in these families was HD – relatives had HD with two reports of over two HD cases within the pedigree. The male predominance in familial cases (3:1) exceeded the male:female ratio of the cases overall (1.5:1) supporting previous observations that familial cases are more common in males than females (Kerzon-Storrar *et al.*, 1983). Unfortunately the study was not able to gather data on male bed-room sharing as a possible explanation for this. There was no consistent age of onset of disease observed within or across families. Examination of the familial relationship suggests that sibling-sibling and parent-child are the most common familial patterns as previously reported (Vianna *et al.*, 1974; Haim, *et al.*, 1982).

This study suggests a possible link between HD and familial MS. Pooled results in the current study identify a near-significant risk, but stratified analyses indicate this is specific to males. The seven cases of MS associated with male HD patients included a mother, two fathers (one of whom also had a sister with MS), the remainder being relatives by marriage rather than direct blood relatives. Recent evidence has implicated an (as yet) unidentified retrovirus in MS (Koprowski *et al.*, 1985).

An association of HD with atopy is demonstrated in this study mainly by the excessive case numbers for eczema/dermatitis, shown in Table VI, but also supported by a significant risk associated with asthma and pollen allergy in young males with NS HD. This was first suggested by Winkelman & Rajka (1982). Such an association was also reported in the previous Yorkshire pilot study (Bernard *et al.*, 1984). However, the relationship to treatment with steroids suggested by our earlier study has not been borne out in this most recent investigation. It should be noted,

**Table II** Hodgkin's disease: Case control study. Topics giving non-significant responses having risk ratios under 2.0 from pooled results.

| | | | |
|---|---|---|---|
| 1 *Past medical history* | | | |
| TB[a] | 6.16 | Malaria | 4.9 |
| Asthma | 15.40 | Personality disorder | 17.28 |
| Any allergy | 73.153 | Migraine | 4.7 |
| Food allergy | 8.18 | Otitis media | 10.13 |
| Fur allergy | 7.17 | Rheumatic fever | 2.11 |
| Pollen/dust allergy | 25.45 | Hypertension | 9.16 |
| Soap allergy | 5.11 | Myocardial infarction | 5.7 |
| Metal allergy | 3.14 | Angina | 1.8 |
| Drug allergy | 29.56 | Pneumonia | 8.9 |
| Other allergy | 22.51 | Duodenal ulcer | 16.27 |
| Reaction to sunlight | 20.46 | Osteoarthritis | 7.14 |
| Bite reaction | 23.58 | Past malignancy | 4.9 |
| Tonsillectomy | 54.131 | Radiotherapy | 5.10 |
| Infectious mononucleosis | 12.23 | | |
| Appendectomy | 34.78 | | |
| Herpes zoster | 11.16 | | |
| 2 *Drug ingestion* | | | |
| Amphetamines | 12.16 | Bronchodilation | 9.28 |
| Contraceptive pill | 39.77 | Steroids | 20.32 |
| Antibiotic | 16.24 | Endocrine | 4.7 |
| Analgesics | 16.33 | Vitamins | 5.7 |
| Antihistamines | 6.11 | Tar based cream | 4.8 |
| Antinausea drops | 2.7 | Migraine tablets | 2.8 |
| Antacids | 10.22 | Drugs for heart disease | 24.47 |
| Benzodiazepines | 33.77 | Anti-inflammatory drugs | 20.32 |
| Other tranquillizers | 25.32 | Hormones | 3.8 |
| 3 *Social aspects* | | | |
| Received higher education[a] | 30.53 | Pet owner | 219.423 |
| Lived on a farm | 14.27 | Household spray user | 156.324 |
| Ever been abroad | 102.271 | Hair spray user | 62.133 |
| 4 *Occupations* | | | |
| Farmers | 21.47 | Painters | 5.15 |
| Miners | 5.15 | Labourers | 2.8 |
| Dye/chemical workers | 16.27 | Transport workers | 27.74 |
| Glass workers | 4.10 | Warehouse men | 6.20 |
| Furnace men | 8.10 | Clerks | 44.61 |
| Electricians | 31.52 | Sales workers | 53.126 |
| Engineers | 62.127 | Service workers | 67.134 |
| Woodworkers | 16.28 | Professional workers | 28.57 |
| Leather workers | 5.6 | Armed forces | 29.62 |
| Textile workers | 30.51 | Nurses | 6.16 |
| Clothing workers | 28.47 | Spinners | 6.19 |
| Food industry workers | 24.64 | Dry cleaners | 6.14 |
| Printers | 6.19 | Sports and recreational | 8.12 |
| Construction workers | 26.49 | | |
| 5. *Industrial* | | | |
| Chemical | 20.34 | Wood workers | 16.37 |
| Petroleum | 5.13 | Hospital workers | 20.41 |
| Agriculture | 29.45 | | |
| 6 *Contact with* | | | |
| Live animals | 33.71 | Fertilizers | 38.55 |
| Dead animals | 18.40 | Spray paint | 20.44 |
| Wood dust | 24.46 | Epoxy glue | 41.75 |
| Solvents | 55.121 | Irradiation | 6.16 |

[a]Numbers indicate total number of cases and control in series in that order.

however, that the link with steroids in the pilot study was accounted for mainly by non Hodgkin's lymphoma (NHL) in the pooled lymphoid malignancies group. Other skin conditions, excluding eczema or dermatitis, also proved to be significantly associated with HD. Urticaria was identified as the strongest risk factor in the group, reaching significance in the 15–35 year old nodular sclerosing (NS) HD subgroup, when sexes were pooled. This has not been identified in previous studies, but is consistent with the immune perturbation hypothesis.

The study has produced interesting observations on past dental anaesthesia. Prior to 1960 general anaesthesia would usually have been achieved with nitrous oxide and a small amount of oxygen. After 1960 this was supplemented with halothane and there has been little major change in gaseous anaesthesia since then. The implication of nitrous oxide in HD aetiology has some biological basis in that it has been suggested that nitrous oxide interferes with vitamin B12 synthesis and has also specific effects on human neutrophils (Nunn & Morain, 1982).

Interest in IM as an EBV-induced lymphoproliferative disease has led to equivocal results on its possible aetiological significance to HD. It has been suggested that the risk, if it exists, may be within three to six years of

**Table III** Hodgkin's disease: Case control study. Topics based on pooled results which resulted in statistically significant low risk ratios.

| *Past medical history* | *N case* | *N control* | *Risk ratio* | *95% Confidence interval* | *P* |
|---|---|---|---|---|---|
| Ever had any operations | 143 | 326 | 0.7 | 0.5–0.9 | 0.02 |
| Sun lamp use for health | 13 | 99 | 0.3 | 0.2–0.5 | 0.001 |
| (Sun lamp use for tanning | 25 | 70 | 1.0 | 0.6–1.7 | 1.00) |
| X-rays for any reason | 225 | 465 | 0.5 | 0.3–0.9 | 0.02 |
| Chest X-ray | 169 | 368 | 0.7 | 0.5–0.9 | 0.04 |
| Fracture X-ray | 133 | 301 | 0.7 | 0.5–0.9 | 0.04 |
| Procedural/investigative X-ray | 44 | 154 | 0.5 | 0.3–0.7 | 0.001 |
| Dental X-ray | 89 | 224 | 0.7 | 0.5–0.9 | 0.02 |
| ('Shoe shop' foot X-ray | 20 | 36 | 1.1 | 0.6–1.9 | 0.37) |
| *Social characteristics* | | | | | |
| Smoker (*vs.* non-smoking related disease in controls) | 134 | 280 | 0.7 | 0.5–0.9 | 0.02 |
| Wine drinker | 25 | 88 | 0.5 | 0.3–0.8 | 0.004 |
| Spirits drinker | 23 | 67 | 0.7 | 0.4–1.0 | 0.04 |

**Table IV** Hodgkin's disease: Case control study. Pooled ages and sexes. Risks associated with family illnesses.

| | *N case* | *N control* | *Risk ratio* | *95% Confidence interval* | *P* |
|---|---|---|---|---|---|
| Lymphoma/leukaemia in family | 16 | 9 | 3.31 | 1.54–7.11 | 0.001 |
| Lymphoma/leukaemia in family (confirmed reports only) | 9 | 5 | 3.72 | 1.32–10.47 | 0.006 |
| Cancer in 1st or 2nd degree relative (confirmed reports only) | 32 | 43 | 1.61 | 0.99–2.62 | 0.06 |
| Brain tumour in 1st degree relative | 7 | 5 | 2.81 | 0.93–8.54 | 0.06 |
| Breast cancer in 1st degree relative | 13 | 17 | 1.15 | 0.74–3.20 | 0.25 |
| Lung cancer in 1st degree relative | 10 | 18 | 0.24 | 0.50–2.42 | 0.48 |
| Multiple sclerosis in family | 9 | 7 | 2.59 | 0.99–6.81 | 0.06 |

**Table V** Hodgkin's disease: Case control study. Risks associated with previous skin conditions.

| | *Males* | | | | *Females* | | | |
|---|---|---|---|---|---|---|---|---|
| | *N case* | *N control* | *Risk ratio* | *P* | *N case* | *N control* | *Risk ratio* | *P* |
| Skin lesion – all except eczema/dermatitis | 22 | 21 | 2.2 | 0.02 | 12 | 16 | 1.6 | 0.26 |
| Urticaria[b] | 5 | 4 | 2.5 | 0.16 | 2 | 0[a] | | |
| Psoriasis | 2 | 4 | 0.9 | 0.98 | 2 | 9 | 0.4 | 0.22 |
| Warts | 7 | 4 | 3.0 | 0.08 | 1 | 1[a] | | |
| Eczema/dermatitis[b] | 23 | 16 | 2.8 | 0.003 | 13 | 19 | 1.4 | 0.38 |
| Steroid treatment for eczema/dermatitis | 9 | 9 | 2.3 | 0.08 | 7 | 6 | 2.5 | 0.10 |
| Other treatment for eczema/dermatitis[b] | 25 | 18 | 3.1 | 0.001 | 15 | 25 | 1.2 | 0.66 |

[a]Insufficient numbers; [b]Medically confirmed records only and [c]Treatment confirmed but not all original diagnoses.

**Table VI** Hodgkin's disease: Case control results. Risks associated with past dental anaesthesia.

| | *Males* | | | | *Females* | | | |
|---|---|---|---|---|---|---|---|---|
| | *N*[a] *cases* | *N*[a] *controls* | *Risk ratio* | *P* | *N*[a] *cases* | *N*[a] *controls* | *Risk ratio* | *P* |
| Any dental anaesthetic | 31 | 86 | 3.1 | 0.003 | 18 | 69 | 0.9 | 0.94 |
| Dental gas only *vs*. never | 24 | 58 | 3.7 | 0.001 | 11 | 47 | 0.9 | 0.76 |
| Dental gas pre-1960 *vs*. never | 16 | 27 | 5.1 | 0.001 | b | | | |
| Dental gas post-1960 *vs*. never | 6 | 19 | 2.7 | 0.08 | b | | | |

[a]Matching ratio 1 case: 3–4 controls (see text) and [b]No case or control responses.

**Table VII** Hodgkin's disease: Case control study. Risks associated with sibship size using large sibships as standard.

| *Number of siblings of case* | *N cases* | *N controls* | *Risk ratio* | *95% Confidence interval* | *P* |
|---|---|---|---|---|---|
| 0 or 1 | 85 | 148 | 1.8 | 1.1–2.7 | 0.02 |
| 2 | 63 | 112 | 1.6 | 0.9–2.6 | 0.06 |
| 3 or 4 | 57 | 110 | 1.5 | 0.9–2.4 | 0.12 |
| 5 or more | 41 | 116 | 1.0 | | |

diagnosis of IM (Munoz *et al.*, 1978). This was weakly confirmed in the present study, the excess risk being confined to males age 15–35 yr within 5 years of IM.

Social characteristics, especially sibship size have been thought of as having an 'infectious agent' interpretation, largely through the work of Gutensohn and Shapiro (1982). The proposed hypothesis is that HD may arise as an unusual and late host response to a common infection, not experienced at an earlier age in singleton or other small families. The data here and in the pilot study lend support to this hypothesis.

The significant risk associated with male barbiturate users was a unique finding. Barbiturates tended to be prescribed for serious psychological or personality disorders and often in conjunction with other drugs.

Finally the negative findings present some problems. Significant negative associations found from smoking find no support in the literature. In one study, heavy cigarette smokers were found to be at risk for HD although alcohol consumption did not influence risk (Paffenbarger *et al.*, 1977). Although smoking is associated with many different malignancies, there is no good evidence to suggest HD is one of them. However, stratification for family size, as a possible correlate of social class revealed that the negative cigarette smoking risk was most marked for the smaller family sizes and had almost disappeared in sibships of more than 4. The negative risk for smoking in male HD was consistent with results from the pilot study. The suggestion that this may reflect a bias in the hospital control group, where smoking-related diseases could be over-represented, was tested by excluding those controls diagnosed with smoking related diseases at time of interview. The negative association with smoking was still significant.

In summary, the results of this case-control analysis lend support to a multi-step model generated in the course of this study, namely the HD occurs largely in those with genetic predisposition, immune perturbation and infectious agent stimulation. These may be thought of independently or as stages in disease susceptibility and may have several possible manifestations in any individual. The concept of genetic predisposition is supported by the excess results in families of solid tumours and lymphomas. Also the link with atopy might have a genetic basis. The possibility of infectious agents was supported by small family size, a link with infectious mononucleosis and the risks associated with MS. Whilst immune perturbation maybe exemplified by occupational risks, skin diseases and dental anaesthesia risks. It is anticipated that multivariate statistical modelling might lend support to these hypotheses and this is intended once the analyses of the other diseases within the study are completed.

This study could not have been accomplished without the assistance of the numerous colleagues within the Yorkshire Region who have allowed us to interview their patients. The work was funded by the Leukaemia Research Fund and we would specifically like to acknowledge the assistance of Mary Brown, Jill Collins, Sylvia Craven, Felicity Ludolf, Ann Mainwaring, Jan Parker, Jane O'Sullivan, Bernice Pearlman, Carole Startin and Brenda Waller who have worked for several years with us on this project. The support of the Yorkshire Cancer Research Campaign and the Yorkshire Regional Cancer Registry is also acknowledged.

**References**

BERNARD, S.M., CARTWRIGHT, R.A., BIRD, C.C., RICHARDS, I.D.G., LAUDER, I. & ROBERTS, B.E. (1984). Aetiologic factors in lymphoid malignancies: A case-control epidemiological study. *Leukemia Res.*, **00,** 681.

BIRD, C.C., LAUDER, I., KELLET, H.S. & 5 others (1984). Yorkshire Regional Lymphoma Histopathology Panel: Analysis of five years' experience. *J. Path,* **00,** 249.

GUTENSOHN, N. & SHAPIRO, N. (1982). Social class risk factors among children with Hodgkin's disease. *Int. J. Cancer,* **30,** 477.

HAIM, N., COHEN, Y. & ROBINSON, E. (1983). Malignant lymphoma in first-degree blood relatives. *Cancer,* **49,** 2197.

HULL, P.J., DELAMERE, I.W. (1978). Familial Hodgkin's disease. *Postgrad. Med. J.,* **54,** 676.

KERZIN-STORRAR, L., FAED, M.J.W., MACGUILLIVRAY, J.B. & SMITH, P.G. (1983). Incidence of familial Hodgkin's disease. *Br. J. Cancer,* **47,** 707.

KOPROWSKI, H., DE FREITAS, E.C., HARPER, M.E. & 7 others (1985). Multiple sclerosis and human T-cell lymphotropic retroviruses. *Nature*, **318**, 154.

MUNOZ, N., DAVIDSON, R.J.C., WHITTHOF, B., ERICSSON, J.E. & DE THÉ G. (1978). Infectious mononucleosis and Hodgkin's disease. *Int. J. Cancer*, **22**, 10.

NUNN, J.F. MORAIN, C.O. (1982). Nitrous oxide decreases motility of human neutrophils *in vitro*. *Anaesthesiology*, **56**, 45.

PAFFENBARGER, R.S., WING, A.L. & HYDE, T.R. (1977). Characteristics in youth indicative of adult onset of Hodgkin's disease. *J. Natl Cancer Inst.*, **48**, 1489.

ROTHMAN, K. & BOICE, J.D. (1979). Epidemiologic analysis with a programmable calculator. NIH Publication no. 79–1649.

VIANNA, N.J., DAVIES, J.N.P., POLAN, A.K. & WOLFGANG, P. (1974). Familial Hodgkin's disease; an environmental and genetic disorder. *Lancet*, **ii**, 854.

WINKLEMAN, R.K. & RAJKA, G. 1982. Atopic dermatitis and Hodgkin's disease. *Acta Dermatol.*, **63**, 176.